# Wheat DNA Methyltransferase TaMET1 Negatively Regulates Salicylic Acid Biosynthesis to Facilitate Powdery Mildew Susceptibility

**DOI:** 10.3390/jof11120876

**Published:** 2025-12-10

**Authors:** Pengkun Ge, Wanzhen Chen, Jiao Liu, Xiaoyu Wang, Cheng Chang

**Affiliations:** College of Life Sciences, Qingdao University, Qingdao 266071, China

**Keywords:** wheat, DNA methyltransferase, *Blumeria graminis forma specialis tritici*, salicylic acid biosynthesis, DNA methylation, histone acetylation, nucleosome occupancy

## Abstract

Powdery mildew disease caused by the obligate biotrophic fungus *Blumeria graminis forma specialis tritici* (*B.g. tritici*) severely affects grain yields and end-use quality of bread wheat (*Triticum aestivum* L.). Uncovering the mechanism underlying the wheat susceptibility to *B.g. tritici* pathogen could contribute to the wheat breeding against powdery mildew disease. Herein, we revealed that the wheat DNA methyltransferase TaMET1 negatively regulates biosynthesis of defense hormone salicylic acid (SA) to promote powdery mildew susceptibility. Overexpression of *TaMET1* compromised wheat resistance against *B.g. tritici* pathogen, while silencing of *TaMET1* led to the SA overaccumulation and enhanced powdery mildew resistance. TaMET1 directly targets the SA biosynthesis activator gene *TaSARD1.* Decreased DNA methylation, increased histone acetylation, and reduced nucleosome occupancy at *TaSARD1* promoter regions were observed in the *TaMET1*-silenced wheat plants, which is associated with activated *TaSARD1* gene transcription. Silencing of the *TaSARD1* and *TaICS1* genes resulted in attenuated SA biosynthesis and dampened powdery mildew resistance in the *TaMET1*-silenced wheat plants. These results implied that DNA methyltransferase TaMET1 epigenetically suppresses the SA biosynthesis activator gene *TaSARD1* by modulating DNA methylation, histone acetylation and nucleosome occupancy, thereby negatively regulating SA biosynthesis and facilitating the powdery mildew susceptibility.

## 1. Introduction

Staple crop bread wheat (*Triticum aestivum* L.) provides about one fifth of dietary energy consumed by humans [1,2]. Increasing demand for wheat grains is driven by the growing global population [1,2]. However, the fungal pathogen *Blumeria graminis forma specialis tritici* (*B.g. tritici*) causes powdery mildew and results in 5–50% loss of wheat production [3,4,5]. The obligate biotrophic pathogen *B.g. tritici* infects wheat aerial organs predominantly via the air-borne conidia and develops the feeding structure, haustorium, to acquire water and nutrients from host cells, and finally forms the microcolony to produce conidia for more infections [3,4,5]. Cultivating wheat varieties with improved powdery mildew resistance is one of the safest and most effective approaches to control the *B.g. tritici* epidemic [3,4,5]. Disclosing the mechanism underlying the wheat powdery mildew susceptibility could contribute to wheat breeding against the *B.g. tritici* pathogen.

As an important epigenetic mark, DNA methylation catalyzed by the DNA methyltransferase mainly takes place at the cytosine in the sequence contexts of CG, CHG and CHH (H=A, C and T) [6]. As summarized by prior reviews, DNA methylation could function in concert with histone modifications and chromatin remodeling to repress gene transcription [6]. In the dicot model plant *Arabidopsis thaliana*, DNA METHYLTRANSFERASE 1 (AtMET1) plays a key role in the DNA methylation essential for plant development and adaptation to biotic and abiotic stresses [7,8,9,10]. For instance, AtMET1 interacts with the Polycomb group protein MEDEA to suppress autonomous endosperm development [9]. Similarly, AtMET1 mediates DNA Methylation to repress light signaling and influences plant regeneration [10]. However, whether and how MET1 regulates wheat disease susceptibility remains unknown.

In this study, we reported that wheat DNA methyltransferase TaMET1 negatively regulates the biosynthesis of the defense hormone salicylic acid (SA) to promote powdery mildew susceptibility. Overexpression of *TaMET1* compromised wheat resistance against the *B.g. tritici* pathogen, while silencing of *TaMET1* led to the SA overaccumulation and enhanced powdery mildew resistance. TaMET1 directly targets the SA biosynthesis activator gene *TaSARD1.* Decreased DNA methylation, increased histone acetylation, and reduced nucleosome occupancy at *TaSARD1* gene promoter regions were observed in the *TaMET1*-silenced wheat plants, which is associated with the activated *TaSARD1* transcription. Knockdown of the *TaSARD1* and SA biosynthesis gene *TaICS1* expression could attenuate the SA biosynthesis and dampen the powdery mildew resistance in the *TaMET1*-silenced wheat plants. These results implied that the DNA methyltransferase TaMET1 epigenetically suppresses the SA biosynthesis activator gene *TaSARD1* by modulating DNA methylation, histone acetylation and nucleosome occupancy, thereby negatively regulating SA biosynthesis and contributing to the powdery mildew susceptibility. These findings revealed the novel link between MET1 and SA biosynthesis in an important monocot crop.

## 2. Materials and Methods

### 2.1. Plant and Fungal Materials Maintenance

Seedlings of the powdery mildew-susceptible wheat cultivar Yannong 999 were cultivated in growth chambers under 16 h light, 20 °C/8 h dark, 18 °C cycle. The virulent *B.g. tritici* was kept on the Yannong 999 seedlings.

### 2.2. Gene Transcription Rate and Expression Level Measurement

Reverse transcription-quantitative polymerase chain reaction (RT-qPCR) was performed as previously described to analyse the accumulation of gene transcripts [11]. The expression level of the *TaMET1* gene was analyzed using primer 5′GTCCACGTTCTCCAGTTAC3′/5′CCGATTTTTCTTGATCTTG3′, and the primers for analyzing *TaSARD1*, *TaICS1*, *TaPR1*, *TaPR2* and *TaEF1* (internal control) genes were derived from a previous study [11]. The transcription rate of the *TaSARD1* gene was analyzed by the nuclear run-on assay, as previously described [11].

### 2.3. Gene Silencing Assay

Fragments of *TaMET1* were amplified using primers 5′AAGGAAGTTTAA CTGTGCTTGCTCTGAATGG3′/5′AACCACCACCACCGTCAAATGGTGGACCTGATACC3′, and cloned into the pCa-γbLIC vector create construct BSMV-*TaMET1*, as described by Yuan et al. [12]. Constructs BSMV-*TaSARD1* and BSMV-*TaICS1* were derived from a previous study [11]. The barley stripe mosaic virus-induced gene silencing (BSMV-VIGS) assay was performed as described by Yuan et al. [12]. For the Agroinfiltration, about ∼0.7 OD_600_ of Agrobacteria resuspension in infiltration buffer (10 mM MgCl_2_, 10 mM MES, pH 5.2 and 0.1 mM acetosyringone) was infiltrated into leaves of *Nicotinan benthamiana* using a 1-mL needleless syringe.

### 2.4. Gene Overexpression Assay

For the single-cell transient gene overexpression assay, coding regions of *TaMET1-2A*, *TaMET1-2B* and *TaMET1-2D* were amplified using primers 5′GGGGACAAGTTTGTACAAAAAAGCAGGCTTC ATGGTGAAAAGTCCACGTTC3′/5′GGGGACCACTTTGTACAAGAAAGCTGGGTCTCAGGCCTGCTGGTGCTTCC3′, 5′GGGGACAAGTTTGTACAAAAAAGCAGGCTTCATGGTGAAAAGTCCACGTTC3′/5′GGGGACCACTTTGTACAAGAAAGCTGGGTCTCAGGCCTGCTGACGCTTCG3′, 5′GGGGACAAGTTTGTACAAAAAAGCAGGCTTCATGGTGAAAAGTCCACGTTC3′/5′GGGGACCACTTTGTACAAGAAAGCTGGGTCTCAGGCCTGCTGGCGCTTCG3′ and PCR products were cloned into the pIPKb001 to create the pIPKb001-*TaMET1-2A* (for OE-*TaMET1-2A*), pIPKb001-*TaMET1-2B* (for OE-*TaMET1-2B*) and pIPKb001-*TaMET1-2D* (for OE-*TaMET1-2D*) constructs. The single-cell transient gene overexpression assay was performed as previously described [11]. A 900-psi dip rupture disc was employed to ensure the helium pressure used, and at least bombardments was conducted for each sample.

### 2.5. Wheat-Powdery Mildew Interaction Analysis

Formation of *B.g. tritici* haustoria and microcolony was statistically analyzed as previously described to determine the wheat-powdery mildew interaction [11]. For the haustorium index (HI%) calculation, at least 50 *B.g. tritici*-infected wheat epidermal cells were analyzed per experiment. For the microcolony index (MI%) calculation, at least 1000 *B.g. tritici*-wheat interaction sites were counted per experiment.

### 2.6. SA Content Analysis

SA content was analyzed by high-performance liquid chromatography (HPLC) as previously described [11]. The free SA amount was descried as ng mg^−1^ fresh weight (FW) with reference to the amount of internal standard ortho-anisic acid.

### 2.7. DNA Methylation Analysis

DNA methylation at *TaSARD1* promoter regions was analyzed by bisulfite sequencing. Genomic DNA samples of 0.5 μg, treated with conversion reagents in the kit, were employed as a template for subsequent PCR. Promoter regions of *TaSARD1.1-6A*, *TaSARD1.1-6B*, *TaSARD1.1-6D*, *TaSARD1.2-6A*, *TaSARD1.2-6B* and *TaSARD1.2-6D* were amplified using primers 5′ATAGGTCTACACATGCACCA3′/5′GCAAGCTAGCTCTAGTCTC3′, 5′AGATAGGACTACACATGTAC3′/5′GATTGATTTCAGTGCACGCA3′, 5′TGGTACATAAACTTCGAAGG3′/5′GCTGCAGGCAGCTAGGGAGA3′, 5′CAATTGAAGAAGACGAAAAC3′/5′AGAGAAATCGCTGAAAGTTA3′ and 5′AAGACGAAGATGTTCTCAAC3′/5′AGGAACAAGCTTGGAACAAG3′. The PCR products were cloned into pGEM-T Easy vector and transformed into bacteria *Escherichia coli*, and more than 25 individual clones for each genotype were sequenced.

### 2.8. Chromatin Immunoprecipitation Assay and Nucleosomal Occupancy Analysis

The chromatin immunoprecipitation (ChIP) assay analyzing the enrichment of TaMET1-HA protein and histone acetylation mark H3K9ac at *TaSARD1* gene promoter regions was conducted as previously described [12]. Briefly, about 300 mg wheat protoplasts or leaves were harvested, and antibodies α-HA (Santa Cruz Biotechnology, Dallas, TX, USA, sc-805) α-H3K14ac (Abcam, Cambridge, UK, ab46984) and α-H3 (Abcam, ab1791) were employed for the ChIP. Relative enrichments of histone acetylation mark H3K9ac were calculated by normalizing the histone acetylation and ChIP with histone H3. Nucleosome occupancy micrococcal nuclease (MNase) assay, analyzing chromatin assembly structure at *TaSARD1* promoter regions, was conducted as previously described [11]. Primer sequences for qPCR analyzing *TaSARD1* promoter were derived from previous studies [11].

### 2.9. Statistical Analysis

For the statistical analysis, at least 5 wheat leaves (for the gene expression, nucleosomal occupancy and histone acetylation at gene promoter regions, cuticular wax and SA levels measurement), 300 mg wheat protoplasts (for the analysis of protein enrichment at gene promoter regions), 50 *Bgt*-infected wheat epidermal cells, and 1000 *Bgt*–wheat interaction sites were analyzed in one experiment or were randomly chosen for each group. At least three independent experiments were performed for each assay and three technical replicates per assay were analyzed using the Student’s *t*-test, and the value represents the mean ± standard deviation (*n.s. p* > 0.05, * 0.01 < *p* < 0.05, ** *p* < 0.01, *n.s.* represents no significant difference). These assays were repeated in three independent biological replicates using dependently prepared samples with similar results.

## 3. Results

### 3.1. Homology-Based Identification of Wheat TaMET1 Genes

**Figure 1:**
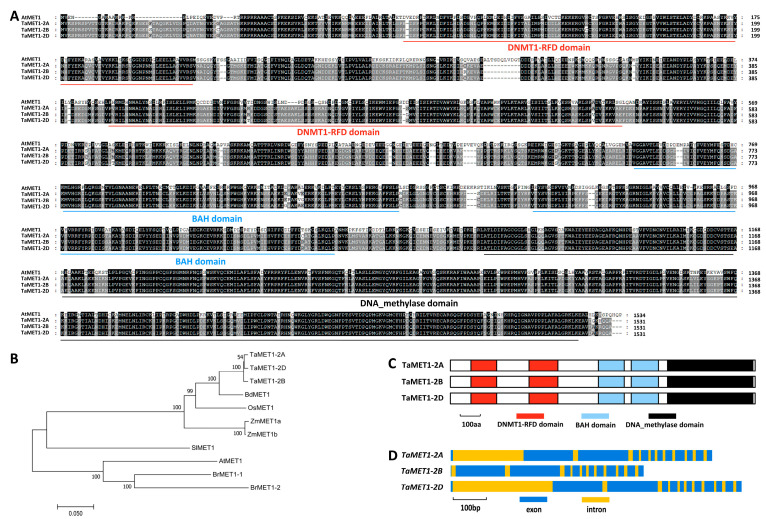
Analysis of wheat TaMET1 sequence and domains. (**A**) Protein sequence alignments of Arabidopsis AtMET1, wheat TaMET1-2A, TaMET1-2B and TaMET1-2D. (**B**) Phylogenetic relationships of the MET1 proteins from Arabidopsis, mustard, tomato. (**C**) Domain arrangement of wheat TaMET1-2A, TaMET1-2B and TaMET1-2D proteins. (**D**) Exon and intron arrangement of wheat *TaMET1-2A*, *TaMET1-2B* and *TaMET1-2D* genes.

In this study, we aimed to explore the putative regulation of DNA METHYLTRANSFERASE 1 (MET1) on wheat’s susceptibility to the *B.g. tritici* pathogen. To this end, we first employed the amino acid sequence of Arabidopsis AtMET1 (At5g49160) as a query to search the reference genome of the hexaploid bread (*Triticum aestivum* L., AABBDD). *TaMET1-2A* (*TraesCS2A02G235900*), *TaMET1-2B* (*TraesCS2B02G260800*) and *TaMET1-2D* (*TraesCS2D02G241800*) were identified from wheat chromosomes 2A, 2B and 2D as AtMET1 homologs. TaMET1-2A, TaMET1-2B and TaMET1-2D proteins shared more than 55% identity with Arabidopsis AtMET1 (Figure 1A). Phylogenetic analysis validated that wheat TaMET1-6A, TaMET1-6B and TaMET1-6D proteins are wheat close homologs of Arabidopsis AtMET1, mustard BrMET1, tomato SlMET1, *Brachypodium* BdMET1, maize ZmMET1 and rice OsMET1 (Figure 1B). As shown in Figure 1C, two Cytosine specific DNA methyltransferase replication foci (DNMT1-RFDs) domains, two BAH domains and one C-5 cytosine-specific DNA methylase (DNA_methylase) domain were identified from all TaMET1 proteins. A total of 12 exons and 11 introns exist in the coding regions of *TaMET1-2A*, *TaMET1-2B* and *TaMET1-2D* genomic sequences (Figure 1D).

### 3.2. Functional Analysis of TaMET1 Genes in the Regulation of Wheat Susceptibility to B.g. tritici

**Figure 2:**
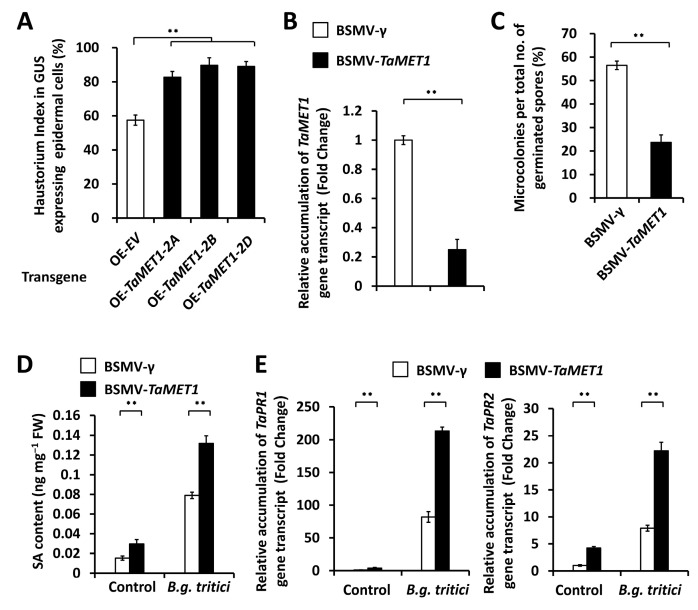
Functional analysis of *TaMET1* gene in the regulation of compatible wheat-powdery mildew interaction. (**A**) Statistical analysis of powdery mildew haustorial formation in wheat epidermal cells transiently overexpressing *TaMET1*. Empty vector (OE-EV) was employed as control and at least 50 *B.g. tritici*-infected wheat epidermal cells were analyzed per experiment (**B**) RT-qPCR analysis of *TaMET1* transcript accumulation in the wheat leaves silencing *TaMET1*. (**C**) Statistical analysis of powdery mildew microcolony formation on wheat leaves silencing *TaMET1*. (**D**) Measurement of SA content in the wheat leaves silencing *TaMET1*. (**E**) RT-qPCR analysis of *TaPR1* and *TaPR2* transcript accumulation in the wheat leaves silencing *TaMET1*. For (**B**–**E**), leaves of wheat plants infected with BSMV-γ were employed as the negative control. For (**A**–**E**), three technical replicates per treatment were statistically analyzed and data are presented as the mean ± SE (Student’s *t*-test; ** *p* < 0.01), and these assays were repeated in three independent biological replicates with similar results.

We overexpressed *TaMET1-2A*, *TaMET1-2B* and *TaMET1-2D* genes in the wheat leaf epidermal cells by bombardment, and inoculated on these bombarded wheat leaves with *B.g. tritici* conidia. Powdery mildew haustorium index (HI%) increased from about 57.6% for the empty vector (OE-EV) control to above 82.7% on wheat cells overexpressing *TaMET1-2A*, *TaMET1-2B*, or *TaMET1-2D* gene (Figure 2A). We silence all endogenous *TaMET1* genes in the wheat leaves using BSMV-VIGS. RT-qPCR confirmed that *TaMET1* gene expression levels decreased significantly in *TaMET1*-silenced wheat leaves (Figure 2B). *B.g. tritici* conidia were inoculated on these BSMV-VIGS wheat leaves, and the formation of *B.g. tritici* microcolony was statistically analyzed. As shown in Figure 2C, the powdery mildew microcolony index (MI%) decreased from 56.4% for the control (BSMV-γ) wheat leaves to 23.6% for *TaMET1*-silenced wheat leaves. These results implicated that the *TaMET1* gene positively regulates wheat powdery mildew susceptibility. The HPLC assay demonstrated that silencing of *TaMET1* gene resulted in the increased SA accumulation level, indicating that *TaMET1* negatively regulates the SA accumulation in bread wheat. Consistently, silencing of *TaMET1* resulted in the enhanced expression levels of SA signaling marker genes *TaPR1* and *TaPR2* (Figure 2E). These results suggested that TaMET1 suppresses SA biosynthesis and positively regulates wheat susceptibility to *B.g. tritici* pathogen.

### 3.3. Epigenetic Regulation of SA Biosynthesis Activator Gene TaSARD1 by TaMET1

**Figure 3:**
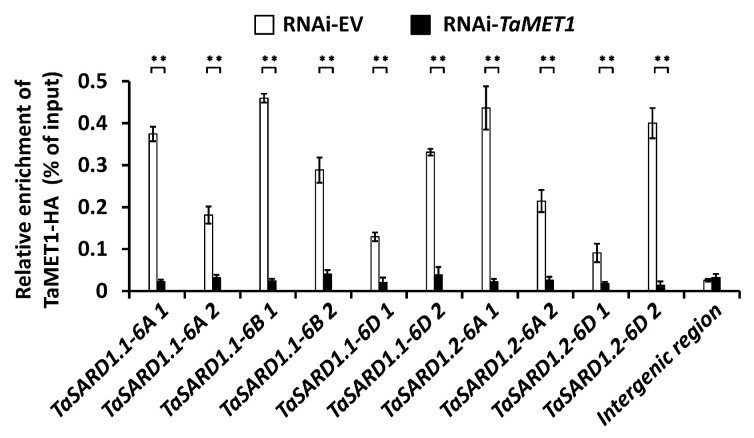
Analysis of the TaMET1 enrichment at *TaSARD1* promoters. ChIP-qPCR analysis of TaMET1-HA enrichment at *TaSARD1* promoter regions in the wheat cells. RNAi-TaMET1 was employed as control. Three technical replicates per treatment were statistically analyzed, and data are presented as the mean ± SE (Student’s *t*-test; ** *p* < 0.01), and these assays were repeated in three independent biological replicates with similar results.

Accumulating studies revealed that SA biosynthesis activator gene *TaSARD1* is regulated by epigenetic events [11,13,14,15]. We ask whether the DNA methyltransferase TaMET1 could target *TaSARD1* gene. To this end, we expressed the TaMET1-HA fusion protein in the wheat protoplasts and performed a ChIP assay to characterize the enrichment of TaMET1-HA proteins at *TaSARD1* promoters (Figure 3). *TaSARD1* promoter regions regulated by epigenetic events like histone acetylation and chromatin assembly were chosen for the ChIP analysis. As shown in Figure 3, these *TaSARD1* promoter regions were found to be enriched in DNA samples co immuno-precipitated with TaMET1-HA protein, indicating that DNA methyltransferase TaMET1 could enrich at promoter regions of the *TaSARD1* genes.

**Figure 4:**
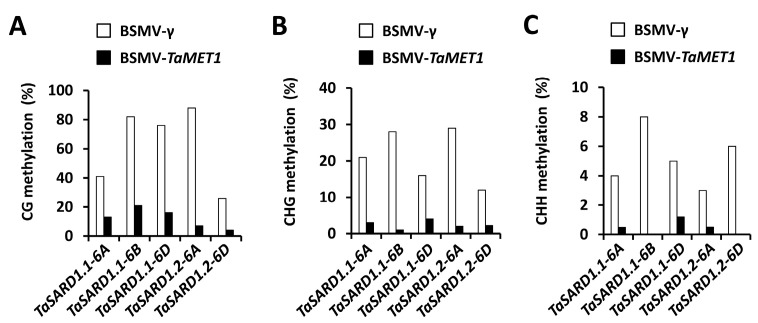
Cytosine methylation analysis of *TaSARD1* loci in *TaMET1*-silenced wheat leaves. Bisulfite sequencing analysis of TaSARD1 loci in *TaMET1*-silenced wheat leaves. The percentage cytosine methylation is indicated for each genotype, as determined at CG (**A**), CHG (**B**) and CHH (**C**) sites for at least 50 clones. H represents A, T, or C. Leaves of wheat plants infected with BSMV-*TaMET1* were employed as the negative control.

**Figure 5:**
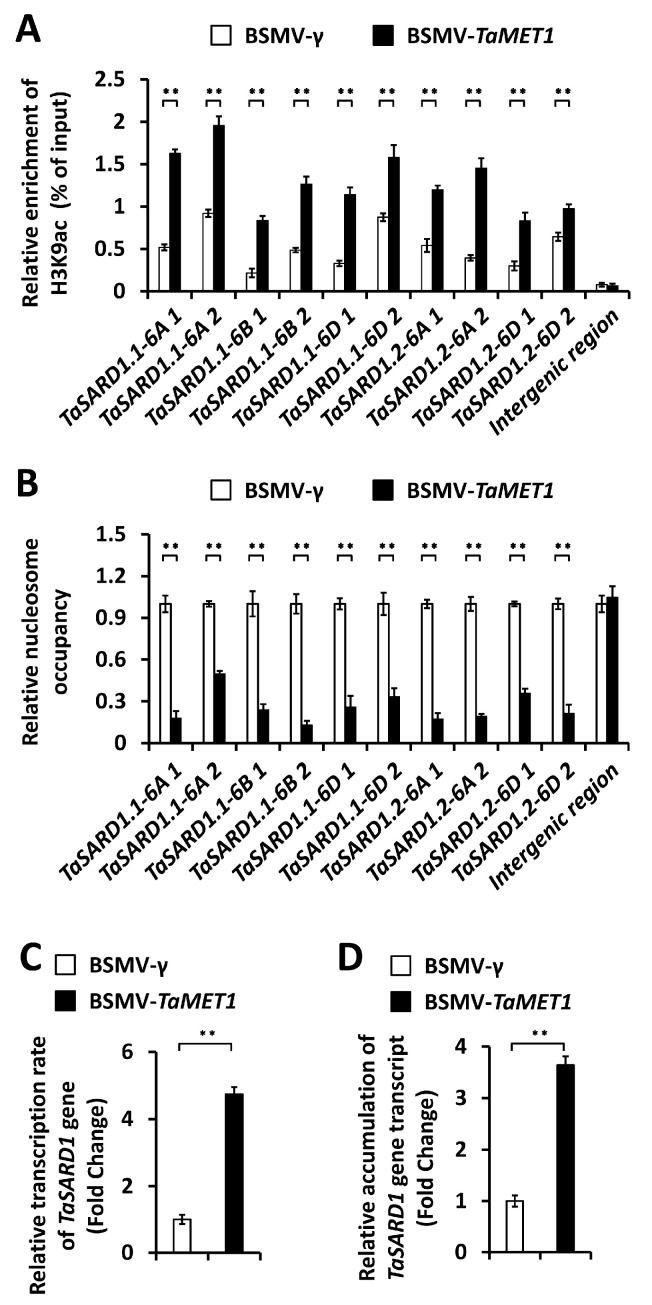
Characterization of histone acetylation, nucleosomal occupancy and gene transcription at *TaSARD1* loci in *TaMET1*-silenced wheat leaves. (**A**) ChIP-qPCR analysis of the H3K9ac abundance at *TaSARD1* promoter regions in the *TaMET1*-silenced wheat leaves. (**B**) MNase analysis of nucleosome occupancy at *TaSARD1* promoters in the *TaMET1*-silenced wheat leaves. The nucleosome occupancy levels in wheat leaves infected with the BSMV-γ empty vector were set to 1.0. Transcription rates (**B**) and expression levels (**C**) of *TaSARD1* gene in the *TaMET1*-silenced wheat leaves were measured by nuclear run-on and RT-qPCR assays, respectively. For (**A**–**D**), leaves of wheat plants infected with BSMV-γ were employed as the negative control, and three technical replicates per treatment were statistically analyzed and data are presented as the mean ± SE (Student’s *t*-test; ** *p* < 0.01), and these assays were repeated in three independent biological replicates with similar results.

To examine whether DNA methyltransferase TaMET1 affects DNA methylation, histone acetylation and nucleosome occupancy at *TaSARD1* promoter regions, we conducted the bisulfite sequencing, chromatin immunoprecipitation (ChIP), nucleosome occupancy micrococcal nuclease (MNase) assay to analyze the chromatin state at promoter regions of the *TaSARD1* genes. As shown in Figure 4, silencing of the *TaMET1* gene resulted in significantly reduced DNA methylation in the context of CG, CHG and CHH (H = A, C and T), at *TaSARD1* promoters. Similarly, the levels of histone acetylation H3K9ac at the *TaSARD1* promoter regions were significantly enhanced in the wheat leaves silencing *TaMET1* gene, compared with the BSMV-γ control (Figure 5A). As shown in Figure 5B, silencing of the *TaMET1* gene led to significantly reduced nucleosome occupancy at *TaSARD1* promoters. Nuclear run-on and RT-qPCR assays demonstrated that silencing of the *TaMET1* gene led to a significantly increased transcription rate and transcript accumulation of the *TaSARD1* gene (Figure 5C,D). These results suggested that the DNA methyltransferase TaMET1 negatively regulates *TaSARD1* gene transcription by modulating DNA methylation, histone acetylation and nucleosome occupancy.

### 3.4. Functional Analysis of TaSARD1-TaICS1-SA Circuit in the TaMET1-Governed Wheat Powdery Mildew Susceptibility

**Figure 6:**
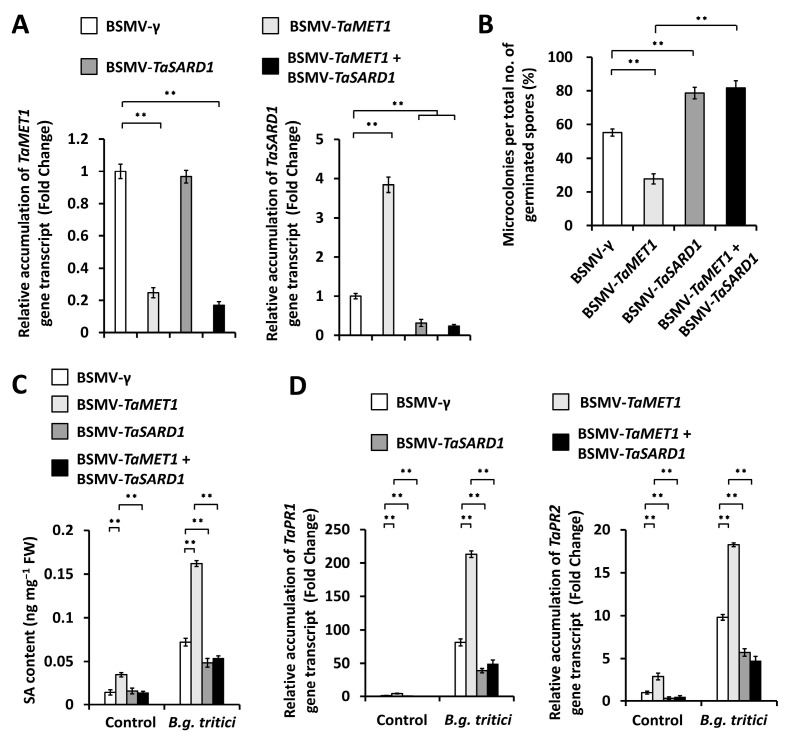
Characterization of genetic interplay of *TaMET1* and *TaSARD1* in the regulation of compatible wheat-powdery mildew interaction. (**A**) RT-qPCR analysis of *TaMET1* and *TaSARD1* transcript accumulation in the wheat leaves silencing *TaMET1*, *TaSARD1*, or co-silencing *TaMET1* and *TaSARD1*. (**B**) Statistical analysis of powdery mildew microcolony formation on wheat leaves silencing *TaMET1*, *TaSARD1*, or co-silencing *TaMET1* and *TaSARD1*. (**C**) Measurement of SA content in the wheat leaves silencing *TaMET1*, *TaSARD1*, or co-silencing *TaMET1* and *TaSARD1*. (**D**) RT-qPCR analysis of *TaPR1* and *TaPR2* transcript accumulation in the wheat leaves wheat leaves silencing *TaMET1*, *TaSARD1*, or co-silencing *TaMET1* and *TaSARD1*. For (**A**–**D**), leaves of wheat plants infected with BSMV-γ were employed as the negative control, and three technical replicates per treatment were statistically analyzed and data are presented as the mean ± SE (Student’s *t*-test; ** *p* < 0.01), and these assays were repeated in three independent biological replicates with similar results.

We ask whether *TaSARD1* gene gets involved in the TaMET1-governed wheat powdery mildew susceptibility. To examine this hypothesis, we conducted the BSMV-VIGS assay to simultaneously silence *TaMET1* and *TaSARD1* genes in the wheat leaves, and found that expression levels of *TaMET1* or *TaSARD1* gene significantly decreased in *TaMET1* and *TaSARD1* co-silenced wheat leaves (Figure 6A). Simultaneous silencing of *TaMET1* and *TaSARD1* genes resulted in the microcolony index (MI%) increased to above 78.7% from 55.2% for the BSMV-γ control wheat leaves (Figure 6B). SA accumulation level significantly decreased in *TaMET1* and *TaSARD1* co-silenced wheat leaves, compared with the BSMV-γ control wheat leaves (Figure 6C). Consistently, simultaneous silencing of *TaMET1* and *TaSARD1* genes resulted in a significant reduction in expression levels of *TaPR1* and *TaPR2* genes, compared with the BSMV-*γ* control (Figure 6D). These data collectively suggested that the DNA methyltransferase TaMET1 negatively regulated SA biosynthesis by epigenetically suppressing the *TaSARD1* gene, thereby facilitating the wheat susceptibility to *B.g. tritici* pathogen.

**Figure 7:**
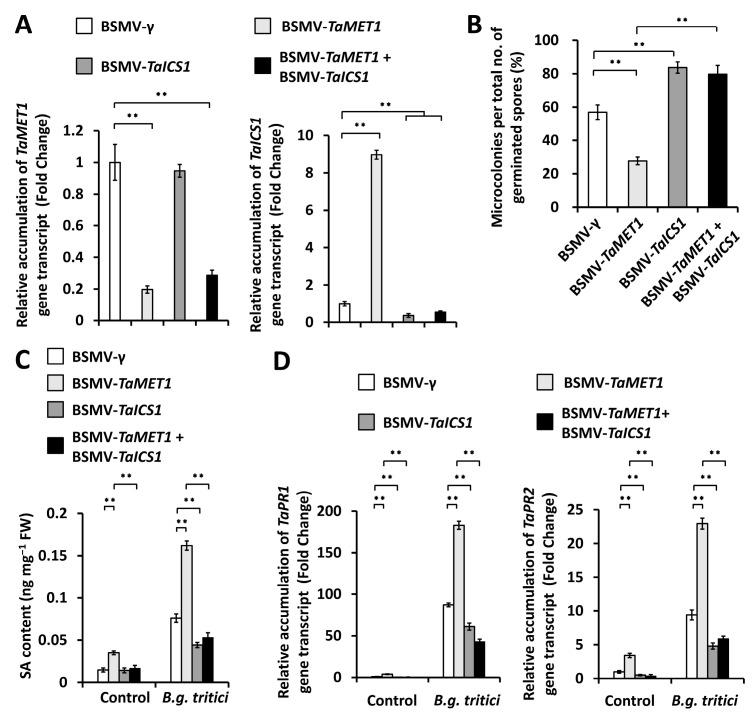
Characterization of genetic interplay of *TaMET1* and *TaICS1* in the regulation of compatible wheat-powdery mildew interaction. (**A**) RT-qPCR analysis of *TaMET1* and *TaICS1* transcript accumulation in the wheat leaves silencing *TaMET1*, *TaICS1*, or co-silencing *TaMET1* and *TaICS1*. (**B**) Statistical analysis of powdery mildew microcolony formation on wheat leaves silencing *TaMET1*, *TaICS1*, or co-silencing *TaMET1* and *TaICS1*. (**C**) Measurement of SA content in the wheat leaves silencing *TaMET1*, *TaICS1*, or co-silencing *TaMET1* and *TaICS1*. (**D**) RT-qPCR analysis of *TaPR1* and *TaPR2* transcript accumulation in the wheat leaves wheat leaves silencing *TaMET1*, *TaICS1*, or co-silencing *TaMET1* and *TaICS1*. For (**A**–**D**), leaves of wheat plants infected with BSMV-γ were employed as the negative control, and three technical replicates per treatment were statistically analyzed and data are presented as the mean ± SE (Student’s *t*-test; ** *p* < 0.01), and these assays were repeated in three independent biological replicates with similar results.

We ask whether *TaICS1* gene encoding a core component of wheat SA biosynthetic machinery gets involved in the *TaMET1*-governed wheat powdery mildew susceptibility [11,13,14,15]. To examine this hypothesis, we conducted the BSMV-VIGS assay to simultaneously silence *TaMET1* and *TaICS1* genes in the wheat leaves, and found that expression levels of *TaMET1* or *TaICS1* gene significantly decreased in *TaMET1* and *TaICS1* co-silenced wheat leaves (Figure 7A). Simultaneous silencing of *TaMET1* and *TaICS1* genes resulted in the significantly increased microcolony index (MI%) (Figure 7B). SA accumulation level significantly decreased in *TaMET1* and *TaICS1* co-silenced wheat leaves, compared with the BSMV-γ control wheat leaves (Figure 7C). Consistently, simultaneous silencing of *TaMET1* and *TaICS1* genes resulted in a significant reduction in expression levels of *TaPR1* and *TaPR2* genes, compared with the BSMV-*γ* control (Figure 7D). These results collectively implicated DNA methyltransferase TaMET1 epigenetically suppresses SA biosynthesis activator gene *TaSARD1*, thereby negatively regulating SA biosynthesis and facilitating the compatible wheat-powdery mildew interaction.

## 4. Discussion

### 4.1. The DNA Methyltransferase TaMET1 Facilitates the Wheat Powdery Mildew Susceptibility

In this study, *TaMET1-2A*, *TaMET1-2B* and *TaMET1-2D* separately located on wheat chromosomes 2A, 2B and 2D were identified as AtMET1 homologs in the hexaploid bread (*Triticum aestivum* L., AABBDD). Transient overexpression of *TaMET1-2A*, *TaMET1-2B* and *TaMET1-2D* in wheat epidermal cells led to the increased haustorium index (HI%), whereas silencing of *TaMET1* resulted in the decreased microcolony index (MI%), suggesting that the DNA methyltransferase TaMET1 positively regulates wheat powdery mildew susceptibility and facilitates the *B.g. tritici* post-penetration events haustorial development and microcolony formation. Histone modifiers and chromatin remodeling factors have been characterized to regulate wheat powdery mildew susceptibility. For instance, histone deacetylase TaHDA6 positively regulates wheat powdery mildew susceptibility, while wheat histone acetyltransferase TaHAG1 negatively regulates wheat powdery mildew susceptibility [16,17]. Similarly, wheat chromatin remodeling protein TaSWP73 facilitates the wheat susceptibility to *B.g. tritici* pathogen [13]. These findings implicated that wheat susceptibility to powdery mildew pathogen is governed by DNA methylation, histone (de)acetylation and chromatin remodeling.

### 4.2. The DNA Methyltransferase TaMET1 Epigenetically Suppresses SA Biosynthesis

Accumulating evidence revealed that defense-related hormone SA plays a key role in wheat powdery mildew resistance [11,13,14,15]. In this study, we demonstrated that DNA methyltransferase TaMET1 enriches at promoter regions of the *TaSARD1*, activator gene of wheat SA biosynthesis, and negatively regulates *TaSARD1* gene transcription by modulating DNA methylation, histone acetylation and nucleosome occupancy. SA overaccumulation and activation of defense marker genes *TaPR1* and *TaPR2* were observed in the *TaMET1*-silenced wheat plants. Notably, silencing of *TaSARD1* and the SA biosynthesis gene *TaICS1* could attenuate the SA biosynthesis and powdery mildew resistance potentiated in the *TaMET1*-silenced wheat plants. These results suggested that DNA methyltransferase TaMET1 epigenetically suppresses SA biosynthesis activator gene *TaSARD1*, thereby negatively regulating SA biosynthesis and facilitating the compatible wheat-powdery mildew interaction. These results allow us to propose a model of how DNA methyltransferase TaMET1 regulates wheat powdery mildew susceptibility. In this model, wheat DNA methyltransferase TaMET1 directly targets the SA biosynthesis activator gene *TaSARD1* to promote DNA methylation, and epigenetically suppress *TaSARD1* gene transcription. Consequently, SA biosynthesis gene *TaICS1* expression and SA biosynthesis is maintained at a resting state, facilitating the wheat powdery mildew susceptibility. In the absence of DNA methyltransferase TaMET1, decreased DNA methylation, increased histone acetylation and reduced nucleosome occupancy were observed at *TaSARD1* promoter regions, which is associated with activated *TaSARD1* gene transcription. Consequently, SA biosynthesis gene *TaICS1* expression and SA biosynthesis is activated, attenuating the wheat powdery mildew susceptibility.

Wheat chromatin assembly factor TaSWP73 and chromatin assembly factor CAF-1 were previously demonstrated to suppress *TaSARD1* gene transcription by enhancing nucleosome occupancy [11,13]. For the first time, this study revealed that DNA methylation governed by TaMET1 gets involved in the epigenetic suppression of *TaSARD1* gene. Therefore, it is intriguing to examine the potential interplay among epigenetic regulators TaMET1, TaSWP73 and CAF-1 in the regulation of SA biosynthesis and wheat powdery mildew susceptibility. In the dicot model plant Arabidopsis, AtMET1 interacts with the histone deacetylase AtHDA6 to maintain transposable element silencing [8]. Interestingly, our previous studies demonstrated that wheat histone deacetylase TaHDA6, resembling TaMET1, negatively regulates expression of SA marker genes *TaPR1* and *TaPR2*, and facilitates the wheat susceptibility to *B.g. tritici* pathogen [16]. Examine the potential interaction between TaHDA6 and TaMET1 and analyzing the potential regulation of TaHDA6 on the *TaSARD1* gene transcription might shed novel light into the epigenetic regulation of wheat SA biosynthesis. In addition, the potential epigenetic impact of TaMET1 on the other defense-related genes and SA-independent immunity remains to be explored in future research.

### 4.3. Potentials of Exploiting Susceptibility Gene TaMET1 in Wheat Breeding Against Powdery Mildew Disease

Herein, DNA methyltransferase gene *TaMET1* was characterized as a new *Susceptibility* (*S*) gene contributing to the wheat powdery mildew susceptibility. Overexpression of *TaMET1-2A*, *TaMET1-2B*, or *TaMET1-2D* in wheat epidermal cells led to increased powdery mildew susceptibility, whereas silencing of *TaMET1* genes resulted in SA overaccumulation and decreased powdery mildew susceptibility. Notably, we demonstrated that DNA methyltransferase TaMET1 directly target the SA biosynthesis activator gene *TaSARD1* and negatively regulates *TaSARD1* gene transcription by modulating DNA methylation, histone acetylation and nucleosome occupancy, suggesting that DNA methyltransferase TaMET1 epigenetically suppresses SA biosynthesis activator gene *TaSARD1*, thereby negatively regulating SA biosynthesis and facilitating the compatible wheat-powdery mildew interaction. For the first time, this study identified the DNA methyltransferase gene as an *S* gene governing plant susceptibility to pathogens.

Previous studies have identified multiple *susceptibility* (*S*) genes contributing to the wheat powdery mildew susceptibility [18,19,20,21,22]. Manipulation of S genes by genome editing techniques could enhance wheat disease resistance [23,24,25,26,27,28,29,30,31,32,33,34]. For instance, genome editing of wheat S genes *TaMLO* and *TaEDR1* by CRISPR-Cas9 and TALENs conferred wheat powdery mildew resistance [26,27,34]. Therefore, exploiting the *S* gene *TaMET1* by CRISPR-Cas9, TALENs, or TILLING techniques might represent a new direction for the wheat breeding against powdery mildew resistance. In Arabidopsis, AtMET1 regulates multiple development events and play important roles in maintaining genome integrity. Therefore, the potential pleiotropic effects of *TaMET1* gene on other important agronomic traits, as well as the possible trade-offs for deploying *TaMET1*-based resistance, should be carefully analyzed before its exploitation in wheat breeding. In addition, epigenetic plasticity regulated by wheat TaMET1 under biotic and abiotic stresses remains to be dissected in future research.

## 5. Conclusions

In this study, we reported that wheat DNA methyltransferase TaMET1 negatively regulates SA biosynthesis to promote powdery mildew susceptibility. Overexpression of *TaMET1* resulted in the enhanced wheat susceptibility to *B.g. tritici,* while silencing of *TaMET1* led to the SA overaccumulation and enhanced powdery mildew resistance. TaMET1 directly targets the SA biosynthesis activator gene *TaSARD1.* Decreased DNA methylation, increased histone acetylation, and reduced nucleosome occupancy at TaSARD1 promoter regions were observed in the *TaMET1*-silenced wheat plants, which is associated with the activated *TaSARD1* transcription. Knockdown of *TaSARD1* and SA biosynthesis gene *TaICS1* expression could attenuate the SA biosynthesis and dampen the powdery mildew resistance in the *TaMET1*-silenced wheat plants. These results implicated that the DNA methyltransferase TaMET1 epigenetically suppresses the SA biosynthesis activator gene *TaSARD1* by modulating DNA methylation, histone acetylation and nucleosome occupancy, thereby negatively regulating SA biosynthesis and contributing to the powdery mildew susceptibility. These findings elucidate the novel regulation of DNA methyltransferase MET1 on the molecular plant-fungal pathogen interaction and provide valuable information for genetic improvement of bread wheat against powdery mildew disease.

## Data Availability

The original contributions presented in this study are included in the article. Further inquiries can be directed to the corresponding author.
